# The Long-Term Effect of Radical Prostatectomy on Erectile Function, Urinary Continence, and Lower Urinary Tract Symptoms: A Comparison to Age-Matched Healthy Controls

**DOI:** 10.1155/2017/9615080

**Published:** 2017-02-05

**Authors:** Badereddin Mohamad Al-Ali, Anton Ponholzer, Herbert Augustin, Stephan Madersbacher, Karl Pummer

**Affiliations:** ^1^Department of Urology, Kaiser-Franz-Josef Spital, Vienna, Austria; ^2^Department of Urology, Medical University of Graz, Graz, Austria; ^3^Kazan Federal University, Kazan, Russia; ^4^Krankenhaus der Barmherzigen Brüder, Department of Urology, Vienna, Austria

## Abstract

*Introduction*. To analyze the impact of radical prostatectomy (RPE) on erectile function and lower urinary tract function in comparison to age-matched healthy men.* Materials and Methods*. Patients who underwent radical retropubic prostatectomy completed questionnaires containing the IIEF-5, the Bristol female LUTS questionnaire, and the International Prostate Symptom Score (IPSS).* Results*. Patients after RPE were included (*n* = 363). Age-matched healthy men (*n* = 363) were included. The mean IIEF-5 of patients aged 61–70 yrs after RPE was 10.4 ± 6.6 versus 18.8 ± 5.3 in the control cohort; the respective values for men aged 71–80 yrs after RPE were 7.2 ± 6.5 versus 13.6 ± 7.7 in the control cohort. Urinary incontinence after RPE was reported in 41.9% (61–70 years) and 37.7% (71–80) versus 7.5% and 15.1% in the control cohort. The mean IPSS of patients after RPE aged 61–70 yrs was 5.0 ± 4.4 versus 5.5 ± 4.9 in the control cohort; the respective values for men aged 71–80 yrs were 6.0 ± 4.9 versus 7.5 ± 5.7 in the healthy cohort.* Conclusions*. The negative effect of radical prostatectomy on erectile and urinary incontinence remains substantial. The physiologically declining erectile and lower urinary tract function with ageing reduces the difference between healthy men and those after surgery. Healthy men have a higher IPSS presumably due to the presence of bladder outlet obstruction.

## 1. Introduction 

Besides the issue of overtreatment, the negative effect of active treatment of localized prostate cancer on lower urinary tract and erectile function is one of the major burdens regarding screening and treatment of prostate cancer [1, 2].

This negative effect of radical prostatectomy (RPE) on lower urinary tract function (erectile function, urinary incontinence) is well documented for two decades, although relevant discrepancies regarding the extent of this effect remain even after this long time period of clinical experience and research [3]. This is exemplified by the reported rates of erectile dysfunction after RPE [4]. Moreover, cohort studies generated by centres of excellence provide potency rates in the range of 90–95%; independent surveys and a recent meta-analysis of the placebo-arms of randomized controlled trials on penile rehabilitation suggest considerable lower rates between 20 and 30% [4, 5]. Similar discrepant data were reported for urinary incontinence [6, 7]. It remains debateable whether the robotic approach provides superior outcome since level I evidence is missing.

Given the life expectancy after RPE, the long-term effect of surgery on lower urinary tract function is of considerable interest and an important parameter for patients, surgeons, and also socioeconomic aspects. To analyze this long-term effect of surgery, the impact of age on lower urinary function has to be taken into consideration [8]. Moreover, to study the long-term effect of RPE on lower urinary tract function two study designs are possible, that is, RCT in comparison to active surveillance/watchful waiting or a matched-pair and cross-sectional comparison to unoperated men. The latter approach was chosen in this study.

Men with a minimum follow-up of 5 yrs after nerve-sparing open retropubic RPE and no adjuvant therapy were compared to age-matched healthy men that were recruited via a voluntary health investigation. All patients completed the International Index of Erectile Function- (IIEF-) 5 [9], the Bristol female LUTS questionnaire [10], and the International Prostate Symptom Score (IPSS).

## 2. Materials and Methods

### 2.1. Study Design

A consecutive series of patients who underwent open uni- or bilateral nerve-sparing retropubic RPE were contacted by surface mail to complete a 10-page questionnaire. For the current study, only men with a minimum follow-up of 5 yrs after RPE and without any adjuvant therapy were eligible.

As a control cohort [11], age-matched men without previous prostate surgery or prostate specific medication (alpha-blockers, 5-alfa-reductase inhibitors, and anticholinergics) who underwent a voluntary health investigation were included. Moreover, the following parameters were routinely evaluated: (1) a clinical medical history, (2) documentation of all concurrent medical therapies, (3) physical examination with documentation of age, weight, height, body mass index, heart rate, blood pressure, echocardiogram, and spirometry, (4) sociodemographic parameters including marital status, cigarette smoking, alcohol consumption, level of education, and physical activity, (5) stress factors, (6) urinalysis, and (7) a blood laboratory evaluation of a total of 14 parameters including kidney and liver function tests, red and white cell counts, low and high density lipoprotein, cholesterol, and glucose. The control group was extracted from this database to match the RPE group regarding age. Institutional board approval was obtained.

### 2.2. Questionnaires

Besides various disease-specific aspects (PSA at diagnosis, histology of the RPE-specimen, PSA-relapse, adjuvant therapy, etc.) the questionnaire contained the IIEF-5, the IPSS, and the Bristol female LUTS questionnaire.

### 2.3. Statistical Analysis

All statistical analyses were conducted using Statistical Package for Social Sciences, version 10.0.7 (SPSS Inc., Chicago, IL) and Primer of Biostatistics, Version 5.0 (McGraw-Hill, 2002). All hypotheses testing was 2-sided with *p* < 0.05 considered to be significant.

## 3. Results

### 3.1. Characteristics of the Study Population

A total of 363 men with a mean age of 71 yrs (range: 61–80 yrs) and a mean follow-up of 7.1 yrs after nerve-sparing RPE (range: 5–13 yrs) entered the study. Tumour characteristics were as follows: PSA 8.5 ± 5.5 ng/mL (0.3–56 ng/mL), pT2, 67%, pT3, 33%, positive surgical margin, 22.9%, Gleason score 6, 48%, Gleason score 7, 41%, and Gleason scores 8–10, 11%. To assess the impact of age, patients were grouped into two age cohorts 60–70 yrs (*n* = 176; 66 ± 2.9 yrs) and 71–80 yrs (75 ± 2.9 yrs; *n* = 187). These patients were compared to 363 age-matched men who underwent a health investigation (60–70 yrs; 64 ± 2.9 yrs; *n* = 257; 71–80 yrs; 74 ± 2.8 yrs; *n* = 106).

### 3.2. Erectile Function

**Figure 1:**
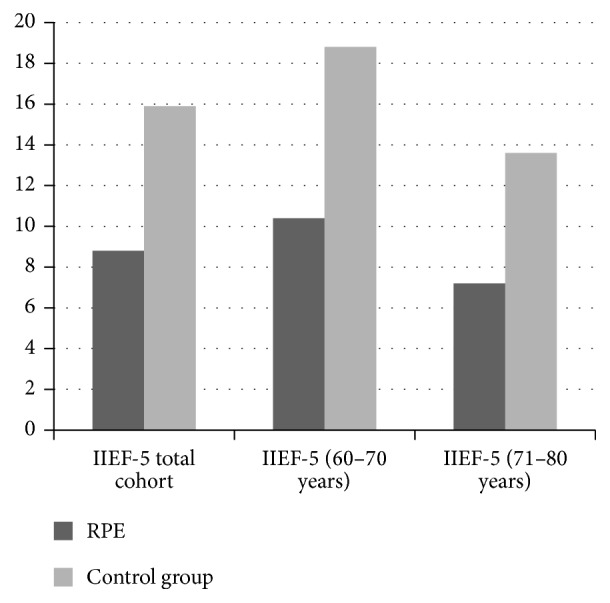
IIEF-5 scores in the RPE and the control group. The differences between the RPE and control cohort were significant for all three groups (*p* < 0.001).

**Figure 2:**
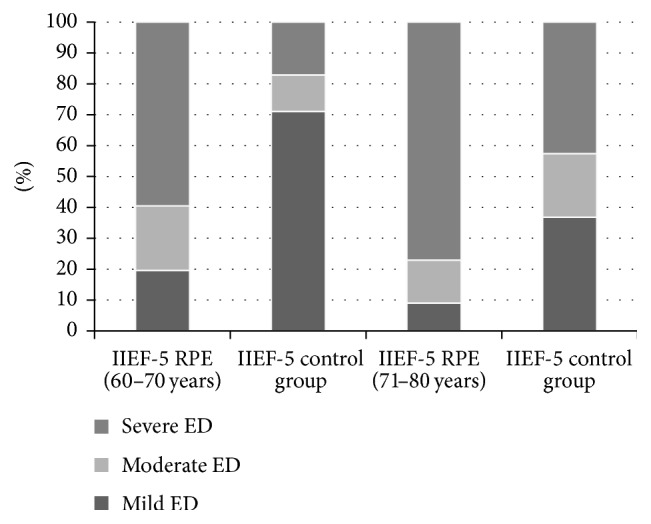
Distribution of mild/moderate/severe erectile dysfunction in the RPE and the control group. The differences between the RPE and control cohort were significant for all three groups (*p* < 0.001).

The mean IIEF-5 of the RPE cohort was 8.8 ± 6.5 compared to 15.9 ± 6.5 in the control group (Figure 1). In patients aged 61–70 yrs after RPE the IIEF-5 was 10.4 ± 6.6 as compared to 18.8 ± 5.3 in the healthy age-matched cohort (Figure 1). The respective values for men aged 71–80 yrs after RPE were 7.2 ± 6.5 versus 13.6 ± 7.7 in the healthy cohort (Figure 1). Moderate to severe ED (IIEF-5 <18) was present in 81% after RPE in both age groups as compared to 17.2% (61–70 yrs) and 37% in those aged 71–80 yrs in the healthy cohort (Figure 2). The risk for moderate/severe ED following RPE compared to healthy men declined from being 4.7-fold in younger age group to being 2.2-fold in the higher age group (Figure 2).

### 3.3. Urinary Incontinence

**Figure 3:**
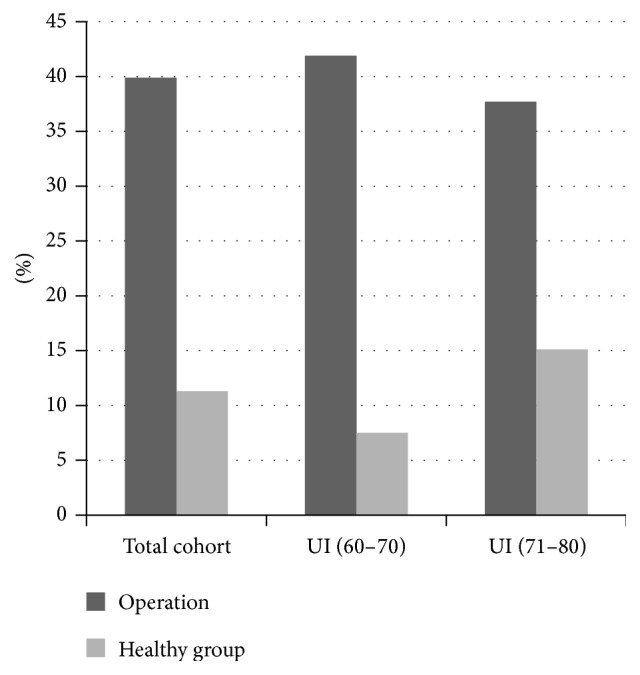
Prevalence of UI in the RPE and the control group. The differences between the RPE and control cohort were significant for all three groups (*p* < 0.001).

The overall prevalence of UI (definition: any involuntary loss during the past 4 weeks) was 39.9% for men after RPE as compared to 11.3% of the healthy cohort (Figure 3). In the 60–70 yrs cohort the prevalence of UI was 41.9% (RPE) and 7.5% (healthy men) and in those aged 71–80 yrs 37.7% (RPE) and 15.1% (healthy men), respectively (Figure 3). The following percentages refer to the number of incontinent patients in each group. Rare episodes of urinary incontinence (once per week or less frequent) were reported after RPE by 54.5% (60–70 yrs) and 36% (71–80 yrs) and in the control group by 27.3% and 18.2%. The respective percentages for more frequent urinary incontinence episodes (≥1/week) were 37.7%, 48.0%, 54.6%, and 45.5%, respectively. Any degree of quality-of-life impairment due to urinary incontinence after RPE was reported by 70.1% (60–70 yrs) and 66.1% (71–80 yrs) and by 81.8% (60–70 yrs) and by 83.3% (71–80 yrs) in the control cohort.

### 3.4. Lower Urinary Tract Symptoms

**Figure 4:**
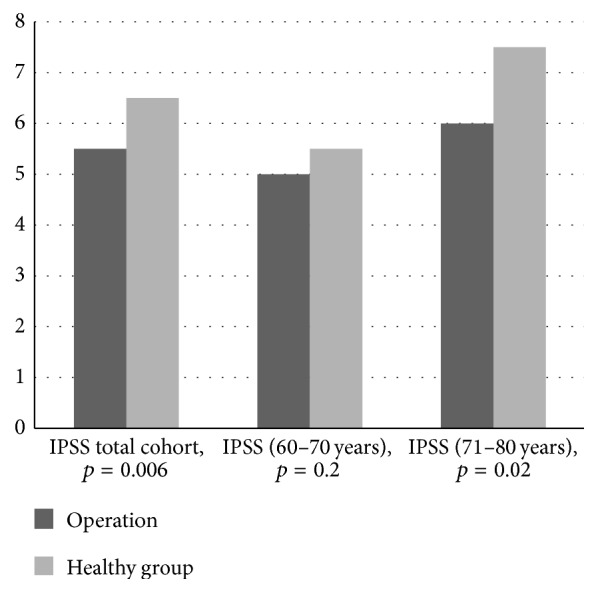
IPSS in the RPE and control group. The differences in the total (*p* = 0.002) and the 71–80 yrs (*p* = 0.02) group were significant.

The mean IPSS was higher in the unoperated group (6.5 ± 5.3) as compared to men after RPE (5.5 ± 4.6) (Figure 4). In the younger age cohort, the IPSS was identical in men who were operated and unoperated on (5.0 ± 4.4 versus 5.2 ± 4.9). In the older age cohort men after surgery had a significantly lower IPSS 6.0 ± 4.9 as compared to unoperated men 7.5 ± 5.7 (Figure 4). Patients in the healthy cohort had higher IPSS scores (IPSS > 8), with a trend towards moderate and severe lower urinary tract symptoms in comparison to surgery cohort. In individuals aged 71–80 yrs, the percentage of men with moderate/severe LUTS increased from 30.8% after surgery to 38.8% in the healthy group.

## 4. Discussion

The aim of our study was to compare the long-term negative effect of RPE on lower urinary tract function to age-matched men participating in a health survey. A comparison to unoperated men might provide a better estimation of the potential long-term negative effect of surgery on lower urinary tract function than a longitudinal cohort study and could be of value in counselling the patient before surgery. To avoid any bias of secondary treatments, only men without adjuvant treatment after RPE were eligible. Moreover, our study had a mean follow-up of >7 yrs after RPE, which is the longest follow-up on this issue. We concentrated on three aspects, that is, erectile function (IIEF-5), urinary incontinence (Bristol LUTS questionnaire), and lower urinary tract symptoms (IPSS).

Reported potency rates after RPE differ substantially [4, 5]. A systematic review by Ficarra et al. [4] showed potency rates 12 months postoperatively from 10 to 73%, 42% to 76%, and 70 to 80% following retropubic, laparoscopic, and robot assisted RPE. Barry et al. [12] investigated 220 patients after open and 406 after robotic RPE. In this cohort patients were at least 66 yrs or older at the time of surgery with only 2.9% after open and 2.3% after robotic surgery reported to have no sexual problems [11]. In a recent meta-analysis of control arms on penile rehabilitation after nerve-sparing RPE Schauer et al. showed that the rate of undisturbed erectile function is in the range of 20–25% in most studies and that these rates have not improved over the past 17 yrs [5]. In our study 61–70 yrs men after RPE had a 4.7-fold higher rate of moderate to severe ED; in the 71–80 yrs cohort this rate declined to 2.2. The only study available with a similar design was reported by Deliveliotis et al. [13] who studied 105 patients after RPE and 80 unoperated control patients recruited in the urological outpatient clinics (follow-up 2 years) [13]. Participants completed various questionnaires, none (with the exception of the AUA-symptoms score) of which was used by us (the validated IIEF-5 was not available then). In this series, erectile function decreased after RPE significantly with only 24.8% of patients having a firm erection compared to 72.8% of the control population [12]. These findings are in agreement with our study.

Similar to erectile dysfunction, the incidence of UI after RPE ranges in the literature between 2.5% and 87% [14]. While some centres of excellence report on continence rates beyond the 90% mark, other sources (e.g., Medicare data) suggest higher incontinence rates [12, 14]. This wide variation is further attributed to definition of UI, methods used for assessing the return of continence, time of reporting after surgery, and patient selection [12, 14]. Herein we used the rather strict definition of the Bristol female LUTS questionnaire, that is, any involuntary loss during the past 4 weeks [10]. By using this definition, already 7.9% of healthy men aged 60–70 yrs and 13.5% in the 71–80 yrs cohort have to be rated as incontinent. In the 60–70 yrs age group, men after RPE had a 5.5-fold higher risk for UI than healthy men; this excess risk declined to be 3-fold in the age group 71–80 yrs. This decline was not due to the lower UI-rates in the surgery group yet to the higher UI in the advanced age control group (13.1%).

The impact of RPE in LUTS was studied in several trials. Schwartz and Lepor performed a prospective study of 104 patients who underwent open radical prostatectomy and reported on the impact after 12 months [15]. In men with moderate/severe LUTS prior surgery, the total AUA symptom score, the symptom problem, and quality-of-life score decreased by 51% (−6.4), 57% (−4.2), and 25% (−0.7) 12 months after RPE [15]. Except for nocturia, all parameters of the AUA symptom score improved significantly [15]. In men with no/mild LUTS no significant changes were observed [15]. Matsubara et al. studied 117 patients after perineal radical prostatectomy and observed a decline of the IPSS in men with moderate/severe LUTS [16]. Wang et al. studied100 consecutive patients after robotic radical prostatectomy with a follow-up of 12 months [17]. The IPSS improved substantially from 14.1 before surgery to 2.9 after 12 months and the IPSS-Ql from 3.4 to 1.6, respectively [17]. Moreover, patients with no/mild LUTS experienced no relevant improvements [17]. Slova and Lepor prospectively followed 453 men for up to 48 months after surgery [18]. The AUA symptom score declined from 6.9 to 5.8 after 4 years, the corresponding numbers for men with an AUA symptom score <8 prior surgery were 3.2 to 4.9; for men with an AUA symptom score >8 the score declined from 13.6 to 7.3 [18]. Storage and voiding symptoms revealed similar patterns [18]. In our cross-sectional study men who were operated on had a lower IPSS as age-matched unoperated men. This underlines the altered natural history of the lower urinary tract in the absence of infravesical obstruction due to the removal of the prostate.

Donovan et al. recently published a series of patient reported outcomes including lower urinary tract and erectile function based on the ProtecT trial [19]. A total of 1643 men were randomized to active monitoring, surgery, or radiotherapy and followed for up to 6 years with several quality-of-life measures [19]. After 6 years (hence a follow-up comparable to our series), the rate of UI and erectile function was—as expected—higher after RPE as compared to active monitoring [19]. The absolute difference between surgery and conservative management, however, was considerably lower in the ProtecT trial as compared to our series [19]. One potential explanation for this discrepancy might be the fact that active surveillance has a negative impact on lower urinary tract function.

## 5. Conclusions

The long-term negative effect of radical prostatectomy on erectile and urinary incontinence remains substantial. The physiologically declining erectile and lower urinary tract function with ageing reduces the difference between unoperated men and those after surgery with advancing age. Unoperated men have a higher IPSS than those after RPE presumable due to the presence of bladder outlet obstruction.
